# A Lecture, Delivered in King's College, London, on Tuesday, the 11th of October, 1831: Being Introductory to the First Botanical Course of the Session Opening the Institution

**Published:** 1832-12

**Authors:** Gilbert T. Burnett

**Affiliations:** Professor of Botany in the College, and Fellow of several Societies.


### BOTANY.

*A Lecture, delivered in King's College, London, on Tuesday, the* 11*th of October,* 1831: *being introductory to the first Botanical Course of the Session opening the Institution.* By Gilbert T. Burnett, Professor of Botany in the College, and Fellow of several Societies.

(Concluded from page 387.)

The herbs and trees (Herbæ et Arbores) of Linnæus, figuratively called by him the Nobles and Elders, (*nobiles et proceres,*) of the vegetable kingdom, and amongst which he distinguished those bearing arms (such as thorns and prickles) as warriors (*milites*) that not only thus defend themselves, but also protect otherwise defenceless vegetables from the aggressions of animals, were collectively denominated by Hill Plants, or Plantæ, under which common name he included all those species which were not reducible to any of his six previous classes, Fungi, Algæ, Musci, Filices, Gramina, et Palmæ. But the plants of Hill thus formed such an extensive group of negatively-characterized and heterogeneous sections, that the term *plant,* instead of being restrained even to the extent that he designed, has long been used as a synonyme for vegetable; and the herbs and trees both of Linnæus and the older writers are so inseparable, and (as systematic groups) so ill-defined, that the words are now indifferently employed, in almost every class, merely to distinguish the larger and perennial from the smaller and more transitory species.

Hence reformation was greatly needed here, and the group has been entirely remodelled and recast; many sections have been excluded, and other classes formed. But, notwithstanding these exclusions, the class still remains very large; yet, though extensive, it is now well characterized and easily defined: for the seeds, instead of being naked, as in the Zamias and the Pines, are invested with a peculiar covering called a pericarp, or seed-vessel, and known commonly as the fruit; such as the fleshy part that is eaten in the melon and the peach, the shell or pod that is thrown away in the nut and the bean. Hence by some botanists these vegetables have been called *Exogenæ Angiospermæ,* or *seed-vesselled,* to contradistinguish them from the *Exogenæ Gymnospermæ,* or

*naked-seeded plants.* Richard and Bartling, who regard other characters as more distinctive and important, call them *Exorhizæ* and *Gymnoblastæ;* but we had rather name them, with special reference to the high development of the fruit and seed, *Fruges,* or *Eucarpæ.* Indeed, this latter change seems necessary, from the exclusion of the plants which form our seventh and ninth classes, which we think improperly blended, whether with the Angiospermæ, Gymnoblastæ, or Exorhizæ, by the botanists who use these names. The gymnospermous Pineales being excluded on the one hand, and the evascular Rafflesias on the other, the *Eucarpæ* or *Cressels,* much as they may differ, and much as they do vary in size and in duration, are mostly coincident in their radiate stratified arrangement, and all in the exogenous disposition of their tubes and cells, by which, with their seeds, they are distinguished from the *Selanthi*; and in the constant development of a seed-vessel at some period of their growth, by which they are known from the Zapini.

Amongst the Fruges, or Eucarpæ, are found many of our culinary vegetables, known commonly as cresses, and some of the most elegant of our garden plants: hence, as a distinction they might familiarly be called *cressels*: *sel,* as in *sel*ago, ground*sel,* &c., indicating worth or beauty.

To illustrate plants so well known seems almost a work of supererogation; and yet not to cite examples from such an important and extensive series might appear to be unpardonable neglect.

Although agreeing in their common and essential characters, in no class is there exhibited a greater diversity in the subordinate developments and the secondary modifications: and hence these plants are distributable, and have been distributed, into very numerous types and sections. These it will be the object of future lectures to describe; it is the general characters of the classes and the regions that, in this introductory conspectus, we chiefly desire to illustrate.

Let, therefore, the oak, the chesnut, and the baobab, as being the most familiar and noble, serve now as sufficient illustrations: for these, besides by their covered fruits exemplifying the class, will, with the fir, the yew, and the cypress of the Pineales, mark most strongly the all but unbounded size to which the Cresc-affines may increase, and the almost indefinite term of their duration.

In my "Amænitates Querneæ" I have collected many records of extreme size and age in trees, especially in oaks; several of these are British, and some are still existing. Perhaps the Tortsworth, the Salcey, the Allouville, and the Cowthorpe, are the most interesting and curious examples: the firstnamed measures fifty-two feet, the second forty-six, and the last, at its lowest level, seventy-eight feet round. Baobabs, however, have been described still larger: Adanson measured several varying from seventy-four to seventy-seven feet in circumference; and Perrottet and Golworthy mention having found them of ninety, and occasionally even exceeding one hundred feet in girth.

The age of several European trees, especially chesnuts and oaks, can be satisfactorily traced by records through many centuries, and of that of more a fair estimate can, in some cases, by other means be formed. Thus, the old chesnut of Tortsworth is known to have numbered above seven hundred, and is calculated to have lived upwards of a thousand years; the age of the Salcey oak has been computed at above a millenium and a half; the oak of Allouville is believed to be between eight and nine hundred years of age; and the Cowthorpe oak is probably more than twice as old. But what then can be the probable age of the still larger baobabs? Of the natural history of these enormous plants too little is at present known to allow any positive deductions to be drawn from their size alone: but still calculations have been made, although not on wholly unexceptionable data. It is with the *degree*, however, that I am less satisfied than even with the *kind* of evidence adduced. And furthermore, I do not think that the observations have been made with sufficient care: for trees increase very irregularly in the several radii of their diametric bulk; and if three hundred rings, measuring, say, three feet across, be granted to be the produce of three centuries on one side of a certain tree, and that an injured or wounded side, this fact cannot alone warrant the conclusion that three feet of the diameter measured on the other unwounded side, or in another tree, have likewise been three hundred years in forming. I do not mean to say that the evidence is bad in principle, but that not enough has been afforded for reducing it to practice: and yet on such evidence it is by some botanists of celebrity asserted, that the smaller baobabs are a thousand, and the middling-sized ones above two thousand, years of age; and hence, forsooth, that the largest which have as yet been found (exceeding one hundred feet in girth,) must have lived for upwards of fifty centuries at least.

But however this may be, it is for the purpose of our present illustrations, a matter of comparatively little moment: for, whether they have numbered quite so many years as their admirers contend or not, their antiquity, doubtless, is extreme, and their sturdy dwarfish stature, as they seldom exceed sixty or seventy feet in height, must favor their almost indefinite duration. Their age, even at the lowest computation, will form a striking example of that one great characteristic of these plants and their allies, the structure of which sets naturally no limit to their duration; and hence it is that they have been called the *Cress-allies*, or *Cresc-affines*.

It must not, however, for a moment be supposed that all the plants included in this region are essentially so long-lived, for many are quite ephemeral; but, however long or short a period they endure, their structure is similar to that of the most long-lived species; and, unlike those included amongst the Term-affines, there is nothing in their structure anatomically incompatible with indefinite duration. But it is time that some further account be given of the examples already cited.

Seven hundred years ago the "great chesnut of Tamworth" was referred to, in writings still extant, as a signal tree; and if, in the reign of King Stephen, A.D. 1135, it was called the Great Chesnut, it is more than probable that it has bounded the manor of Tamworth, now Tortsworth, for upwards of a thousand years. Some time since it measured fifty-two feet in circumference: from calculations that have been made it is believed that in its youth it must have been contemporary with the Saxon Egbert. I have lately made inquiries concerning the state of this venerable tree, and also of the chapel-oak of Allouville, and learn with satisfaction that they are not only still alive, but flourishing in their green old age; and, from the vigour their shoots evince, they will probably outlast the present generation.

The oak of Allouville, in Normandy, known there as the Chêne Chapelle, and to which reference has been made, was, above a century and a quarter since, converted into a place of worship: its trunk was at that time hollow, and its head in part decayed. This living cavern was then paved and roofed, and divided by a floor into two apartments. The lower was fitted up by the Abbé du Détroit as a chapel, and the upper as a dwelling for the officiating priest. The caverns in hollow oaks are, however, seldom devoted to such honourable purposes: that in Damery's, for years, was used as a tavern; in the prison oak at Kidlington, vagrants and other slight offenders are said to have been occasionally confined; and the shell of the venerable Salcey patriarch, which is nearly half as large again as the chapel oak, was formerly enclosed by gates on either side, and cattle penned within it: and inside the hollow of the Cowthorpe oak, it is said that upwards of seventy persons have been at one time assembled. Were it not, as I elsewhere have observed, for instances such as have been mentioned, some of which occur in our own country, and in our own or in our father's time, we might almost be allowed to treat as fables the tales of modern travellers, who tell of hollow trees converted into tombs and prisons, as well as those older histories which declare that the ancient Germans had castles of oak; that in one vast Cerrus a hermit built his cell and chapel; and that another "served both as a castle and a fort." Of these stupendous oaks the history would almost seem to be as monstrous as their reported bulk; but that a hollow oak might be sufficiently large for a hermit's cell and chapel, we have existing proof in the oak of Allouville; and it may be also well conceived, when we reflect that the cavity in Damery's oak was three feet wider than the parish church of St. Lawrence in the Isle of Wight, and that the trunk of the Cowthorpe oak, just noticed, where it meets the ground, stands on a plot exceeding by more than six feet *the length*, and by two feet twice *the width*, of the parochial church just mentioned.

Few persons, indeed, save those to whom habit has rendered it familiar, form any thing like just estimates of the actual size of trees. The situations in which they commonly are seen harmonizing with the illimitable expanse of heaven, and the wild extent of forest scenery or of mountain heights, lessen ideally their apparent bulk. Nor is it, till singled from the surrounding landscape, nor even then until the theodolite or rule proclaim their sums, that we become persuaded of their vast extent: nay, figures themselves, to the generality of the world, convey but very imperfect conceptions of length and breadth, and height and girth; some more familiar representations are wanted to prove that a majestic tree, which is only in moderate proportion as an ornament to nature, in the country, is really an enormous mass, and would be esteemed a large and glorious structure, amongst the dwellings and palaces of men in town. It is by comparing these forest kings with more homely objects, that we alone become acquainted with their correct capacity. When seeing an oak seven feet in diameter, its size arrests not our attention; we even pass with little thought such as hold ten or twelve feet across, or more, although the smallest of these has a width as great as the carriage-way of Fetter lane near Temple bar, or of Bedford street in the Strand. Oaks can be named which would suffer two broad-wheeled waggons to pass each other on the kerf; the stub of one has been described, on which two men could thresh without incommoding each other; and this was not one of the largest size. The chapel oak of Allouville, not half so large as our Cowthorpe tree, is of equal size with the famous Greendale oak, the trunk of which is pierced by a road, over which it forms a triumphal arch, higher by several inches than the entrance to Westminster Abbey (the Poet's Postern), and under which men on horseback pass, and through which carriages have been driven.

The area occupied by the Cowthorpe oak where the trunk enters the soil, exceeds the groundplot of that majestic column of which an oak is confessed to have been the prototype, viz. SMEATON's Eddystone lighthouse. Sections of the stem of the one would, at several heights, nearly correspond with the sections of the curved and cylindrical portions of the other. A chamber of equal extent, or larger, than either of those in the lighthouse might be hollowed out of its trunk; the natural caverns in Damery's and other oaks were larger than the chambers alluded to; and transverse slices of the stem would be considerably too large to floor any of them. Arthur's round table, which is a plank from such an oak, would form for it an entire roof or projecting capital; indeed, upon this table there might be built a round church as large as that of St. Lawrence before referred to, and space to spare; so that if the extent of the sapwood were added, or the groundplot of the Cowthorpe oak were substituted for the table, there would be plenty of room, not only to build the parish church, but also to allow enough for a small cemetery beside. Indeed, with reference to this last-named oak, and also the tree-castles and tree-chapel, I would merely observe that St. Bartholomew's, in the hamlet of Kingsland, between London and Hackney, which, besides the ordinary furniture of a place of religious worship, viz. desks for the minister and clerk, altar, staircase, stove, &c., has pews and seats for one hundred and twenty persons: upwards of one hundred have been in it at the same time; and some months since myself made one of a congregation there assembled of nearly eighty persons (seventy-six or seventy-seven were counted), when the pews were by no means crowded, and plenty of room left vacant: still this chapel is nearly nine feet less in width, and only seventeen inches more in length, than the groundplot of the Cowthorpe oak: in fact, the tree occupies upwards of thirty square feet more surface than does the chapel. Or, to take another illustration; in Little White Lion street, Long Acre, within five minutes' walk of the place where we are now assembled, the inspectors of a district-visiting society found, about nine months ago, a house, the internal area of which is only twelve feet by twenty-four, (not half that of the Cowthorpe oak (which is twenty-six feet in diameter,) containing nine small rooms, in which there dwelt, i.e. ate, drank, and slept, and did all that poor mortality requires, no less than eleven men, thirteen women, and sixty-nine children, making a total of ninety-three human beings, to whom there has been denied, in this great town, so much surface of the earth as is granted for the growth of a single tree.

The next and concluding class is formed by the Selanthi, or Selworts: these plants, like the Pineales, were until lately blended with the Fruges or Eucarpæ, but, although still less as yet are known, those which have been discovered and examined constitute a group as structurally distinct, or even more so, than the Zamiæ and Pini: for in them the vegetable kingdom, after proceeding through many successive stages of development, from seedless to flowering plants, exhibits a return to the point whence the series of evolutions began. They seem, in fact, to form the descending links which connect the highest with the lowest grades of vegetable development.

By their flowers, sometimes evolved in a most exuberant degree, they establish their connexion with the highest flowering plants; while, by their destitution of tubular vessels, and their frequently fungoid characters, they shew their close affinity to the lower mushroom sections. Some of these plants, such as the Cytinus, &c. have long been known, and, from their paradoxical structure, regarded as anomalies and exceptions in the class to which they were formerly referred. But the most splendid of the group, and that which alone would justify their collective name of Selworts, or Selanthi, was only discovered in the year 1818 by Dr. Arnold, the naturalist, who accompanied Sir Stamford Raffles in one of his journeys into the interior of Sumatra. It is said that the natives call it Ambun Ambun, or Krûbût, i. e. the *great flower*; and it is in truth a vegetable Titan. The specimen first found by the lamented Arnold, in remembrance of whom, and likewise of Sir Stamford Raffles, it has been called the Rafflesia Arnoldi, measured *a full yard across;* the petals being twelve inches long and a foot apart from each other; the nectary, adds the Doctor (in an unfinished letter to a friend, which was published posthumously), would, in the opinion of us all, hold *twelve pints*; and the weight of this prodigy we calculated to be fifteen pounds.

Several other allies have since been found, some of which are figured by Blume in his Flora Javæ, but none have been as yet discovered that equal Arnold's flower in bulk.

All these curious plants agree in several particulars. In the first place, they have no proper roots of their own, and they derive their nourishment from the vegetables on which they grow; in the second, they have no stems, the flowers being sessile on the vines that bear them; thirdly, they are destitute of leaves, the buds being covered only by scales, which are purplish or brownish, and resemble the chaffy scales of other parasitic plants; for, as they derive their nourishment already prepared by the leaves of another vegetable, they do not require any foliage of their own: so that here we have plants consisting of flower only, neither root, stem, nor leaves, being truly present. And what seems still more curious is, that, although the largest and most magnificent flowers in the world, they have very little in common with other flowering plants. They have no proper seeds, but are multiplied by *spores,* similar to the spawn of mushrooms, to which indeed their general form and internal structure bear no slight resemblance. The petals are of a mushroom-like substance, and smell like tainted beef; and in them flies deposit their eggs, as they often do in fungi. Again, they contain no tubular or spiral vessels, like most other flowering plants, but consist of cells alone, like the mushroom tribes; they also spring from beneath the bark of the Cissus, which becomes gradually enlarged by their growth, somewhat resembling that false covering which several of the fungi have that grow on living plants; raising the outer surface into tumors, and bursting it as they become more fully developed; such as the blights and blasts of corn, and so forth: and hence these stupendous flowers, which are from six to nine feet in circumference, shew a likeness to the most lowly of the mushroom tribes, many of which are so minute as scarcely to be visible to the naked eye.

The Helosis and Balanophora, with the Cynomorium, or Fungus Melitensis, formerly guarded with such jealous care by the knights of Malta, and sent by the grand master to all the friendly sovereigns and potentates of christendom, as one of the most precious offerings he could make, may be cited as further examples of this extraordinary group. So fungus-like are some of these vegetable paradoxes, that they have been commonly considered such; and the latter, especially, has long been known as *the Maltese champignon,* or the *mushroom of Malta;* and, were it not for the development of stamens and pistils, the propriety of changing the name, and disturbing the old arrangement, might well be questioned. As it is, the characters will fully justify their segregation, as a class, from other flowering plants; the higher grades of which are by them allied to the lower flowerless and leafless sections.

Thus, having traced the gradual evolution of the vegetable organismus, from the simplest of the flags and fungi, through the numerous stages of development which characterize the several orders and regions of the vegetable reign, to the plants included in this final class, the present sketch is closed; for they descend to those with which the series was begun: and, connecting the extremes of an extended scale, declare that however, for convenience, art may reduce the productions of nature to isolated groups, and divide them into separate sections, still that they are divisible by art alone, and, although relatively distinguishable, they are not absolutely separable: for, however diverse the distant members may appear, they are all intimately connected, and essential to each other: and form, in their respective subordinations. but integral parts of one majestic and harmonious whole.

Gentlemen: This bird's eye view of the vegetable kingdom, which has now been condensed into a single lecture, has of necessity been very brief and general: it was, indeed, intended as nothing more than an index or an outline, a sketch or diagram, of the several chief departments, that are recognized or easily recognizable by all. But, slight as the notice of the several classes purposely has been, still here our conspective view must cease; for time forbids any further demonstration: nor is it needed, as hereafter each class must be examined separately and in detail; and it is hoped that the object proposed has already been attained, viz. to prove by actual illustrations what "a varied thing a vegetable is."

Although differing from the usual schemes of introduction, I have thought it advisable thus to begin with a demonstration of plants as they exist; to shew their positive characters, before comparisons are instituted between them and other kingdoms of the organic and inorganic worlds: in fact, first to have materials to compare, before comparative views are taken. Hence, in this first section of our extended course I propose to follow out this plan of demonstrating the special structures, functions, properties, and uses of each succeeding group, from the lowest to the highest grades; before any general views or comparisons are instituted, even between the varied developments of equivalent organs, as pervading the whole vegetable kingdom; and much before any are made between the different, and often essentially diverse, constitutions of the adjacent animal and inorganic reigns. I know that this is not the usual scheme of study, but I am convinced, (and I speak from no slight experience,) I am convinced that its advantages are great, and that it should be occasionally pursued.

This course will, therefore, be devoted to the special study of plants as the subjects of botany; the next to the general investigation of their attributes, as its objects; and the third, or summer section of the series, to a practical application (especially during the herborizing excursions and demonstrations) of those special and general principles, the theories or philosophic views of which have, in the two former portions, been explained. Thus I hope to be enabled to offer to the King's College students, during each session, a complete course of botanical instruction. The subordinate particulars of this scheme I shall not now attempt more fully to develop: enough has perhaps been already said to shew the route that in this autumn term will be pursued: and further observations on the objective view will be more opportunely made at the commencement of the objective series of these lectures, to be delivered in the spring; and to that period I therefore shall reserve them.

But, gentlemen, it is not to plants alone that even in a botanical course I would have you confine your whole attention. Philosophy has lost much of its interest and power by the too, the far too-exclusive dedication of the mind to some one branch alone; and it is only now that knowledge is again raising itself to the state of science from the condition of art, by entering on a more liberal and expanded sphere of exertion.

Gentlemen, recollect that natural history and natural philosophy are, in truth, the history and the philosophy of nature, and not the partial study of some especial parts. Just as philosophy itself does not consist merely of the philosophy of nature or the learning of the schools, but comprises both: it is abstract as well as experimental, intellectual as well as physical. These studies have been too long and too widely severed; let us hope that this College may be the happy means of their re-union: the spirit of the age is favorable to the attempt, and the object is worthy our most strenuous exertions. How eloquently did Ray lament the separation; I cannot forbear quoting his expostulation, his reproof. "We content ourselves (says he), we content ourselves with the knowledge of tongues, and a little skill in philology, or history perhaps, and antiquity, and neglect that which to me seems more material: I mean natural history and the works of the creation. I do not discommend or derogate from those other studies; I should betray mine own ignorance and weakness, should I do so; I only wish they might not altogether jostle out and exclude this. I wish that this might be brought into fashion amongst us; I wish men would be so civil as not to disparage or deride and vilify those studies which themselves skill not of, or are not conversant in: no knowledge can be more pleasant than this, none doth so satisfy and feed the soul; in comparison whereto, that of words and phrases seems to me insipid and jejune." This is just the spirit with which I wish our students to become embued; just the spirit with which I wish them to prosecute their studies; just the spirit with which I shall endeavour to make botany a part of general philosophy, and shew them that the science does not consist in the mere knowledge of names and systems, in the bare ability of deciding on the identity of a species, but also in the equally, if not more important, study of the general laws which regulate the evolutions of their organs, and determine their properties and powers; as well as of the still more general connexions of all the sciences with each other: and with this same spirit, gentlemen, it is that, in the words of my Lord BACON, the *minister et interpres naturæ*, I shall invite you to approach and peruse this volume of the creation, which is written in the only language that hath gone out unto all the ends of the earth, unaffected by the confusion of Babel."

